# Acquired Glucose-6-Phosphate Dehydrogenase Deficiency

**DOI:** 10.3390/jcm11226689

**Published:** 2022-11-11

**Authors:** Giovanni Mario Pes, Maria Pina Dore

**Affiliations:** 1 Dipartimento di Medicina, Chirurgia e Farmacia, University of Sassari, Clinica Medica, Viale San Pietro 8, 07100 Sassari, Italy; 2 Sardinia Longevity Blue Zone Observatory, 08040 Ogliastra, Italy; 3 Department of Medicine, Baylor College of Medicine, One Baylor Plaza Blvd, Houston, TX 77030, USA

**Keywords:** G6PD deficiency, acquired disease, oxidative stress, endocrinopathies

## Abstract

Glucose-6-phosphate dehydrogenase (G6PD) deficiency is a hereditary condition caused by mutations on chromosome X and is transmitted by a sex-linked inheritance. However, impairment of G6PD activity may result from biochemical mechanisms that are able to inhibit the enzyme in specific clinical conditions in the absence of a structural gene-level defect. In this narrative review, a number of clinical settings associated with an “acquired” G6PD deficiency, phenotypically undistinguishable from the primary deficiency, as well as the mechanisms involved, were examined. Hyperaldosteronism and diabetes are the most common culprits of acquired G6PD deficiency. Additional endocrine and metabolic conditions may cause G6PD deficiency in both hospitalized and outpatients. Contrary to the inherited defect, acquired G6PD deficiency is a condition that is potentially curable by removing the factor responsible for enzyme inhibition. Awareness regarding acquired G6PD deficiency by physicians might result in improved recognition and treatment.

## 1. Introduction

**Figure 1:**
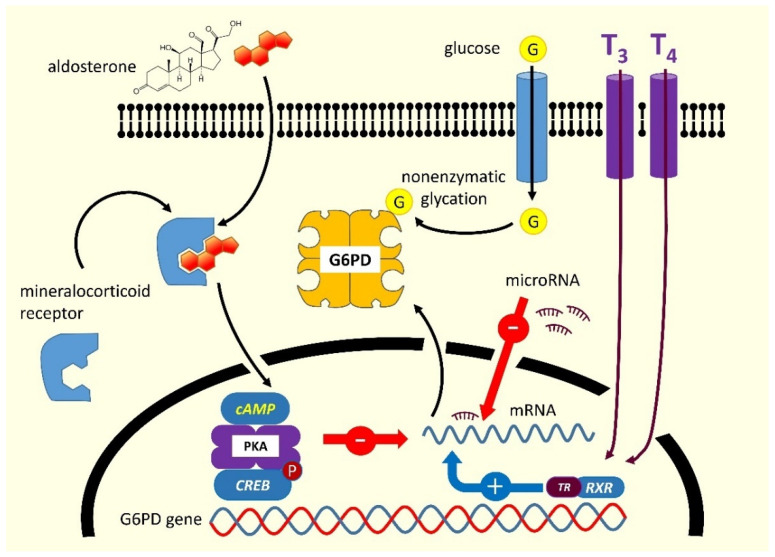
Regulation of G6PD activity and potential inhibitory mechanisms. Aldosterone downregulates the expression of G6PD mRNA through the stimulation of the synthesis of cyclic adenosine monophosphate (cAMP), that binds the tetrameric form of Protein Kinase A (PKA). PKA phosphorylates the transcription factor cAMP response element-binding protein (CREB), thereby eventually blocking the transcription of the *G6PD* gene. Thyroid hormones bind to the thyroid receptor (TR), forming a heterodimer with the retinoid receptor (RXR) and activating G6PD mRNA transcription.

Glucose-6-phosphate dehydrogenase (G6PD) deficiency is the most common human enzyme disorder worldwide, occurring more frequently in malaria-endemic areas [1]. This condition is responsible for neonatal jaundice and or hemolytic anemia following exposure to a number of drugs, infections, or plants containing oxidizing agents [2]. In most cases, the disorder is inherited due to mutations in the gene encoding G6PD (OMIM 305900), mapping on the telomeric region of the distal arm of the X chromosome, and transmitted by a sex-linked mechanism [1,3,4]. The human G6PD enzyme is found in the cytoplasm of all cells. It catalyzes the first reaction of the pentose-phosphate pathway, thereby providing the ribose-5-phosphate necessary for DNA synthesis, and the nicotinamide adenine dinucleotide phosphate (NADP^+^/NADPH), the main hydrogen donor in the biosynthetic reactions [2]. The enzyme is involved in maintaining the intracellular glutathione level and contributing to counteracting reactive oxygen species [5]. Although G6PD can generally be found in all tissues, its deficiency essentially manifests in the red blood cell, wherein, differently from other nucleated cells in the body, no alternative biochemical pathway can ensure NADPH production. The G6PD exists as a monomer, dimer, and tetramer but only the dimer is catalytically functional, and the interconversion between the three forms is critical for enzyme activity [6]. Each subunit has a glucose-6-phosphate binding site, an NADP^+^ catalytic site, and an allosteric NADP^+^ binding site. Any alteration of these structural or regulatory domains, induced by genetic mutations or in the absence of them through binding with external agents can modify the efficiency of catalysis, in some cases severely decreasing it. Furthermore, the influence of endocrine factors that reduce *G6PD* gene transcription is capable of determining an acquired form of decreased G6PD activity [7,8]. Mineralocorticoids downregulate the expression of G6PD mRNA by stimulating the synthesis of cyclic adenosine monophosphate (cAMP), which binds to the tetrameric form of protein kinase A; the latter phosphorylates the transcription factor cAMP response element-binding protein (CREB), thereby blocking the *G6PD* gene transcription. Thyroid hormones bind to the thyroid receptor (TR), which forms a heterodimer with the retinoid receptor (RXR), thereby activating the transcription of the G6PD mRNA. Finally, *G6PD* is a target gene of several microRNAs that play an important role in enzyme regulation acting mostly by suppressing the translation of G6PD mRNA [9,10] (Figure 1).

In addition to the involvement in acute hemolysis, G6PD deficiency has recently been reappraised as a risk factor for a broader spectrum of diseases with inflammatory pathogenesis, such as cardiovascular disease [11,12], asthma [13], or celiac disease [14], probably resulting from inflammasome activation [15]. Most cases of G6PD deficiency are genetically transmitted non-modifiable primary disorders, and their prompt recognition may avoid exposure of carriers to agents that are potentially hemolytic. On the other hand, acquired forms of G6PD deficiency, usually transient, and unrelated to genetic defects have been described [16,17,18]. Similar to the patients with hereditary G6PD deficiency, those with acquired forms are vulnerable to oxidative damage, which is usually short-lasting and treatable by removing the underlying cause. Clinicians should recognize the existence and non-negligible frequency of these acquired forms of reduced G6PD efficiency to provide patients with appropriate therapy.

## 2. Factors Potentially Inducing Acquired G6PD Deficiency

Acquired forms of G6PD impairment may be difficult to diagnose, because of the need of ruling out the presence of mutations in the gene structure. In fact, genuine acquired forms are those in which biochemical mechanisms make an intact enzyme molecule that is less efficient through different pathways. Thus, it is not surprising that in the literature, few cases of acquired forms of reduced G6PD activity have been described, since, in most instances, the presence of genetic mutations is neither routinely screened nor definitively excluded [19]. In fact, some of the case reports where deficiency was tested in blood and no mutation was found, might be explained by technical limitations including the lack of a full *G6PD* gene sequence. In the section below, enzyme impairment arising from non-genetic extrinsic mechanisms reported in the literature and summarized in Table 1 is critically discussed.

### 2.1. Blood Disorders

Early studies in large cohorts of patients with blood disorders described acquired forms of enzymopathies, particularly G6PD deficiency, with a frequency of nearly 3% [44]. Enzyme deficiency, apparently unrelated to any demonstrable *G6PD* gene mutation and detectable in most tissues, was described in a few case reports. Recently, in a Libyan patient with chronic myelomonocytic leukemia, persistent hemolytic anemia was observed in the absence of obvious extracorpuscular causes [18]. Despite a lowered G6PD activity (21.3% residual activity), the genomic DNA sequence, obtained from the hair bulb, did not reveal the presence of mutations. In contrast, the DNA sequence from white blood cells revealed a novel nonsense variant predicted to generate a non-functional truncated protein. This “acquired” G6PD deficiency was not the result of genomic DNA mutations, but rather a somatic mutation arising in a specific clone of blood marrow cells [18]. Similarly, patients who have received G6PD-deficient stem cells from unaware donors and subsequently found to be enzymopenic can be presumed to have developed an acquired form of G6PD deficiency. One case reported by Au et al. underlined the importance of carefully monitoring the integrity of the *G6PD* gene in potential hematopoietic stem cell transplant donors, as well as recipients who should be provided with the same medical attention as individuals with primary G6PD deficiency [20]. Infrequently, an acquired form of G6PD deficiency can originate from transfusions of deficient red cells from donors not previously screened for the enzyme activity [21,22]. In some cases, the activity reduction may be so massive, or the volume of transfused blood so large, that it causes clinically significant hemolysis in the recipient. In populations with a high prevalence of G6PD deficiency, it is advisable to assess G6PD in potential blood donors, according to the WHO guidelines. Deficient donors should be discouraged to donate blood. Moreover, blood bags containing G6PD-deficient red cells should not be used for intrauterine transfusion, neonatal exchange transfusion, or for G6PD-deficient patients [45].

### 2.2. Ingestion of Chemicals

It is well known that the ingestion of a number of substances, chemicals, or medicines is potentially able to elicit a hemolytic crisis in subjects with a predisposing G6PD defect. However, there is also evidence that several molecules can induce a significant reduction in the enzyme catalytic activity without evidence of genetic transmission of G6PD mutations [18,46]. Their diagnosis is challenging, as G6PD mutations are not routinely screened, except in a few populations with a high frequency of the disorder [47]. However, case reports have been published describing successfully treated hemolytic episodes without significant reduction in G6PD activity in the blood, thereby raising the suspicion of a transient acquired form [18,44,48,49]. The ingestion of specific chemical substances may significantly disrupt G6PD activity and, rarely, cause massive hemolysis. In some of these cases, the chemical culprit may have triggered a latent genetic predisposition to enzymatic insufficiency, thereby causing impairment of antioxidant defense, notably on the erythrocyte membranes. Yet, other cases might arise due to the direct action of the chemical agent on the enzyme protein. A report by Hulshof et al. described a case of sodium chlorite intoxication, which caused severe hemolysis in the absence of predisposing G6PD mutations [17]. This situation was associated with severe methemoglobinemia which appeared to be a key component in the pathophysiology. The case described was treated by the administration of methylene blue, which has a reducing effect on methemoglobinemia and should also be considered as a supporting treatment in similar cases of acquired G6PD deficiency. Despite their rarity, these cases suggest that in patients with hemolytic anemia, in addition to the common laboratory tests, it is mandatory to collect a comprehensive medication history including the use of herbal supplements and phytotherapeutics of traditional Chinese and Ayurvedic medicine [23].

### 2.3. Endocrine Disorders

#### 2.3.1. Excess of Mineralocorticoids

The inhibition of mammalian G6PD by C17- and C20-ketosteroids has been known since the early 1960s [24]. In contrast, steroids possessing a hydroxyl rather than a ketone group in C17 or C20, such as corticosteroids, estrogen, and progesterone, have little or no inhibitory effect on G6PD [24]. It has been demonstrated that inhibition by ketosteroid is uncompetitive, which is a rare occurrence in the case of substances different from the enzyme’s substrates [50]. In vitro, acquired G6PD deficiency has been described in association with the excess of ketosteroid precursors secreted by the adrenal gland [51]. Dehydroepiandrosterone (DHEA, also known as androstenolone, 3β-hydroxyandrost-5-en-17-one, or 5-androsten-3β-of-17-one) is an important endogenous steroid hormone [52]. This molecule, and its sulfate conjugate (DHEAS), display the highest serum concentration among androgens. The DHEA is a potent non-competitive inhibitor of G6PD activity [53,54]. Since its action is mainly targeted at healthy erythrocytes, it reduces the corpuscular glutathione thereby making parasitization by *Plasmodium falciparum* more difficult [55] and, for this reason, it has been used to treat malaria [56]. The excess of adrenal androgens in the pathogenesis of acquired G6PD deficiency was also studied in animal models of polycystic ovary syndrome [30]. Aldosterone, an adrenal hormone structurally similar to DHEA, is a potent inhibitor of the G6PD activity. Hyperaldosteronism and other conditions related to an excess of mineralocorticoids, promote oxidative stress, and impair endothelial function and vascular reactivity by decreasing G6PD activity [31,51]. On the contrary, gene transfer of *G6PD* in animal models improves vascular reactivity by overexpressing G6PD, thereby confirming the abovementioned pathogenetic connection. The mechanism by which aldosterone inhibits G6PD has been investigated using both in vitro and in vivo models, especially in endothelial cells. The hormone increases the levels of cAMP, which interacts with the tetrameric form of protein kinase A (PKA) by dissociating its regulatory subunits from the catalytic ones. The PKA in turn inhibits the expression of CREB/CREM transcription factors that are directly involved in the G6PD transcription [51] (Figure 1). In line with these findings, it has been hypothesized that an acquired and reversible form of G6PD deficiency can be induced by excess mineralocorticoids, thus eventually impairing the antioxidant defense of cells. This hypothesis was confirmed by the restoration of normal vascular reactivity by removing the G6PD-deficient state via mineralocorticosteroid receptor blockade [20]. In addition, since the use of natural thiols such as α-lipoic acid has been shown to be beneficial in the treatment of individuals with congenital G6PD deficiency [57], it is likely that supplementation with this or similar compounds may prove useful in balancing the redox status in subjects with acquired G6PD deficiency as well.

#### 2.3.2. Hypothyroidism

In vitro models have shown that thyroxine (3,3′,5,5′-tetraiodothyronine—T4), the main product of the thyroid gland, can activate the G6PD enzyme by competing with NADPH for the same binding site, despite the lack of structural similarity between the two molecules [25,26]. In vivo models also provided evidence of G6PD inhibition following decreased thyroid function. For instance, in rats made hypothyroid through a combined treatment with propylthiouracil and iopamide, the G6PD activity was reduced by 28%, while the administration of T2 restored the activity [27,28]. The administration of 3,3′,5′-triiodothyronine (T3) reversed the effects in thyroidectomized rats. In another study performed on rodents, a significantly decreased G6PD activity was observed after thyroidectomy, whereas hyperthyroidism increased both enzymes [29]. These findings further confirm the hypermetabolic effects of thyroid hormones on cellular metabolism. Although the majority of the physiological effects of thyroid hormones are mediated by the c-erbA family of nuclear receptors, there is evidence that thyroid hormones can upregulate various enzymatic activities independently of protein synthesis via non-nuclear mechanisms. This effect has been investigated mostly in the liver tissue, but it was also observed in the brown adipose tissue, which is the one with the highest lipogenic potential in the body [29]. The decreased activity of thyroid function is biologically relevant and produces a significant G6PD inhibition and, in turn, a greater tissue vulnerability to stressors [58]. An adequate thyroid function can protect against oxidative stress [59]. In humans, the findings are less common, yet G6PD deficiency associated with congenital hypothyroidism has been described in Iranian newborns [32]. Interestingly, G6PD levels returned to normal after 120 days of treatment with levothyroxine, thereby indicating that the deficiency was not of genetic origin.

#### 2.3.3. Diabetes

In experimental animals, the injection of insulin increases the activity of G6PD by fivefold, and this effect is amplified by the co-administration of glucocorticoids [7,60,61]. The induction of enzyme activity probably depends on the increased rate of protein synthesis as demonstrated by immunotitration experiments using anti-G6PD antibodies [7]. It follows that, in some cases, insulin deficiency could be associated with reduced G6PD activity. In streptozotocin-induced diabetes in mice, G6PD activity in the liver was reduced by about 50%, especially in the presence of concomitant copper deficiency [38]. A recent study by Parsanathan et al. revealed that treatment of human aortic endothelial cells with high glucose or palmitate decreases G6PD activity and increases inflammatory cytokines and cell adhesion molecules, suggesting another plausible mechanism of acquired G6PD deficient status [62].

Evidence from the literature, including both case reports [33,34,35] and clinical surveys [63], has shown that G6PD deficiency is more frequently found in patients with diabetes compared with the general population. However, other studies did not confirm these findings [64]. In many cases, it was possible to demonstrate the co-existence of mutations within the *G6PD* gene [35,65]. Nonetheless, in rare cases, a consistently lower G6PD activity has been described in diabetic patients without a detectable genetic mutation.

Early studies have suggested that, rather than diabetes itself, it is the ketoacidosis that triggers hemolytic episodes, although only in the presence of a pre-existing state of G6PD deficiency [66,67,68]. It has been speculated that insulin infusion to correct the ketoacidotic state may reduce glucose availability with subsequent NADPH depletion and impaired antioxidant capacity, specifically affecting β-cells [69]. More recently, it has been proposed that acquired forms of G6PD deficiency in diabetes may be precipitated by metabolic decompensation driven by severe hyperglycemia. In such cases, enzyme inhibition does not depend on insulin regulation of G6PD synthesis, but instead on the increased glucose levels that promote non-enzymatic glycation of the enzyme protein, therefore providing a mechanistic explanation of the progressive loss of catalytic activity [70]. A further contribution to the G6PD inhibition may be related to the increased glucagon level, which is always present to some extent in diabetes, given that it is known that the injection of glucagon into experimental animals reduces G6PD activity [71]. Moreover, pancreatic β-cells are very vulnerable to oxidative stress [72], and depletion of antioxidant capacity due to any cause, innate or acquired—including G6PD deficiency—severely impairs insulin secretion, thereby creating a self-sustaining loop.

A high frequency of decreased G6PD activity, apparently unrelated to gene mutations, has been reported in ketosis-prone diabetes, an atypical form of diabetes common in male descendants of sub-Saharan Africans, primarily African American and African Caribbean [36,73]. A study revealed a high prevalence (up to 42.3%) of G6PD deficiency without coding or intronic G6PD mutations in patients suffering from this form of diabetes [37], although the impact of potential technical issues such as poor storage or degradation of samples cannot be totally ruled out. Interestingly, the severity of G6PD deficiency was positively correlated to the magnitude of insulin deficiency, thereby further confirming that normal G6PD activity is necessary to preserve β-cell function [37]. However, in this form of diabetes, G6PD deficiency is unlikely to be the sole result of the metabolic decompensation, since experimentally-induced hyperglycemia by infusion in subjects with this form of diabetes did not inhibit erythrocyte G6PD activity [36].

#### 2.3.4. Obesity

Adipose tissue displays the highest expression of G6PD after blood [74]. According to some studies, the upregulation of this enzyme in adipocytes is involved in the pathogenesis of metabolic syndrome [74,75]. Increased enzyme activity in fat tissue is usually interpreted as a response to inflammation and oxidative damage that characterizes this condition. However, the enzyme is not increased in the plasma of obese subjects, indicating that its dysregulation is tissue-restricted. Acquired G6PD deficiency has been observed in inflammatory disorders associated with metabolic syndrome. The study by Gheita et al., which recruited 40 cases of rheumatoid arthritis and 30 cases of Sjögren syndrome, reported a frequency of G6PD deficiency of 87.5% and 30%, respectively. In the former case, the level of catalytic activity was significantly lower in patients with concomitant metabolic syndrome [19]. Clearly, these high frequencies cannot be explained by the presence of structural defects of the coding gene and are quite indicative of an acquired form.

### 2.4. Preeclampsia

Preeclampsia indicates the onset or worsening of blood hypertension accompanied by proteinuria that develops in females after the 20th week of gestation and presents with seizures unrelated to other causes [76]. The study by Afzal-Ahmed et al. provided evidence that preeclamptic pregnancies are associated with reduced G6PD activity, NADPH depletion, and impairment of the redox balance in erythrocytes and fetal endothelial cells [39]. In this disorder, the reduced/oxidized glutathione ratio is half as compared to normotensive pregnant females [40]. Although some cases of preeclampsia have been linked to a mutation in the *G6PD* gene [77], it should be considered an acquired form of G6PD deficiency in most cases.

### 2.5. Micronutrient Deficiency

Since the 1950s, it has been known that the activity of G6PD in the liver decreases in animals subjected to calorie restriction and increases in the re-feeding phase, especially if the diet is rich in carbohydrates [78,79,80]. Human individuals with protein-calorie malnutrition, or deficiencies of specific micronutrients, show a lower expression of G6PD, which can potentially be restored by adequate nutrition. Furthermore, a study conducted on Filipino children, investigated the levels of zinc in deficient and non-deficient G6PD babies, finding significantly lower values (60% vs. 82%) in the former [43]. This observation highlights that the deficiency of certain micronutrients may be a predisposing factor for acquired forms of G6PD deficiency occurring in malnourished subjects, although it cannot be excluded that the deficiency of trace elements, necessary to adequately respond to oxidative challenge, could unmask a previously unknown congenital G6PD deficiency.

Vitamin D deficiency can also alter the activity of G6PD. The biologically active form of vitamin D, or 1,25-hydroxycholecalciferol, is able to enhance G6PD levels by interacting with the vitamin D receptor (VDR) which is a transcription factor binding a Vitamin D response element (VDRE) in the first intron of the coding gene [81,82]. In rats fed a low-vitamin D diet, the G6PD activity appears to be reduced in the liver and kidney [42]. In the duodenum of vitamin D-deficient rats, G6PD was 60% lower than in controls [41]. In vitro, vitamin D is able to upregulate G6PD in a dose-dependent manner [81]; in vivo, vitamin deficiency might be associated with reduced antioxidant defense due to the depletion of intracellular concentration of reduced glutathione [31]. In case of unexplained hemolysis, the clinician should suspect vitamin D deficiency which, if proven, should be treated by supplemental therapy.

## 3. Discussion

**Figure 2:**
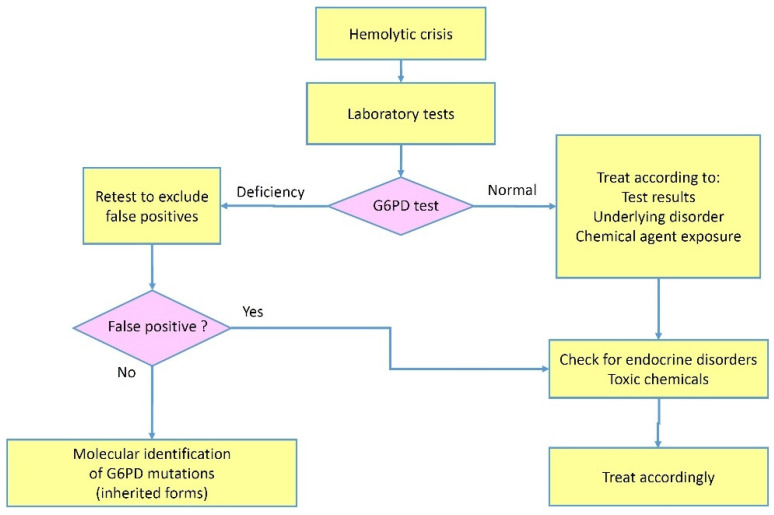
Flowchart of the diagnostic process in case of suspected acquired G6PD deficiency.

The G6PD deficiency, one of the most common enzymopathies reported worldwide, is usually regarded as genetically determined by hereditary defects in the *G6PD* gene. Although there have been early reports of “acquired” forms in the literature [16,44,48,49], in which genetic mutations were thoroughly ruled out, these conditions have received little attention due to their rarity and the difficulty in undertaking an accurate search for a potential genetic defect. However, the short duration of their clinical manifestations, and the lack of evidence of familial predisposition, make these forms nosologically different from the usual hereditary conditions. In the most severe cases, acquired forms are revealed by a hemolytic crisis arising in subjects with an underlying disease, often an endocrine (diabetes, primary hyperaldosteronism) or inflammatory (rheumatoid arthritis) imbalance. In other cases, the condition is milder and does not manifest with hemolytic crises but may be incidentally discovered during routine laboratory tests or for research purposes. A typical scenario might involve a patient who develops hemolytic episodes, in which laboratory findings show a temporary reduction of the G6PD catalytic activity that cannot be confirmed later. In these cases, the disorder displays all the features of a transitory condition independent of genetic causes and reasonably ascribable to the underlying disease (Figure 2).

Based on experimental data, the mechanisms involved in acquired G6PD deficiency can be summarized as follows:

- G6PD is physiologically regulated by several hormones whose release may change in the course of certain diseases, such as primary hyperaldosteronism, diabetes, and hyperglucagonemia, responsible for the majority of acquired forms, producing a temporary post-transcriptional G6PD downregulation;
- Chemical substances may act directly as inhibitors of G6PD: accidental or voluntary ingestion of such substances can result in a hemolytic crisis undistinguishable from that occurring in inherited forms;
- In a number of hematological diseases, the presence of unknown circulating G6PD inhibitors (hormones? microRNAs?) has been postulated [46]. Experiments in which the plasma of these patients was incubated with red blood cells from healthy donors showed a significant decrease in the activity of many enzymes, therefore confirming the presence of an inhibitory agent [48]. However, this inactivating mechanism was demonstrable only in a limited percentage of cases.

Once the diagnosis of hemolytic anemia is established, it is important to ascertain the cause using information from the history, physical examination, and directed laboratory testing. According to the reported causes of acquired G6PD deficiency in the literature, a test indicating a G6PD activity below the normal range requires carefully ruling out a false positive test. In female patients, a positive test could theoretically stem from excessive suppression of X chromosome inactivation in the blood lineage, especially in elderly women. In general, a qualitative test, e.g., a fluorescent spot test under ultra-violet light, in our opinion, is not recommendable in favor of a quantitative biochemical test. The catalytical rate should be adjusted for the hemoglobin levels and the reticulocyte count. When a transfusion is planned, for reasons of urgency, a screening test may be used, but it should be followed by a quantitative confirmatory test as soon as possible. If the suspicion of an acquired form of G6PD deficiency remains despite the confirmatory test, the test should be repeated at least 120 days after hemolytic episode resolution (average time required for the replacement of circulating red blood cells) to check whether the deficiency status is permanent or transient. False-negative tests may occur due to the co-presence of heterozygous β-thalassemia (frequent in Mediterranean populations or in other populations characterized in the past by endemic malaria). The differential diagnosis should be extended to the so-called acquired hemolytic anemias due to a number of immune and non-immune causes. These conditions can display all marks of hemolysis but G6PD is usually normal. In the case of an underlying endocrine disease, in which the hormone involved is known to potentially affect the G6PD expression, the patient should be managed, when possible, by correcting the underlying endocrinopathy. Finally, in the presence of an acquired G6PD deficiency, physicians should avoid administering unsafe drugs acting as oxidative stressors that can precipitate hemolysis.

This review may have a few caveats. The major limitation is that up to date the literature has reported very few cases of acquired forms of G6PD, and in a few studies, the precise etiology of the acquired condition was not identified. Additionally, it is possible that some cases were missed by the authors, thereby making the list incomplete. Nonetheless, this narrative review collected the most significant studies in the field, providing for the first time, a comprehensive overview of acquired G6PD deficiency, which is still an underestimated problem, especially in clinical practice.

## Figures and Tables

**Table 1:** List of studies reviewed according to the target (in vivo, in vitro, and in humans) and factors involved in acquired G6PD deficiency.

Clinical Condition	In Vitro Studies	In Vivo Studies	Studies in Humans
Blood disorders	–	–	Somatic mutation in bone marrow progenitor cells [18]; hematopoietic stem cell transplantation [20]; transfusion from deficient donors [21,22]
Ingestion of chemicals	–	–	Sodium chlorite [17] Herbal supplements [23]
Endocrine disorders	Excess of mineralocorticoids [24] Hypothyroidism [25]	Hypothyroid state by using drugs [26,27,28], Thyroidectomy [29] Polycystic ovary [30]	Excess of mineralocorticoids [31] Congenital hypothyroidism [32] Diabetes [33,34,35] Ketosis-prone diabetes [36,37]
		Streptozotocin-induced diabetes [38]	Rheumatoid arthritis associated with metabolic syndrome [19]
Preeclampsia	–	–	Impaired redox status [39,40]
Micronutrient deficiency	–	Rat duodenal mucosa [41]; vitamin D deficiency [42]	Zinc deficiency [43]

## Data Availability

Not applicable.
